# Urban malaria in sub-Saharan Africa: a scoping review of epidemiologic studies

**DOI:** 10.1186/s12936-025-05368-9

**Published:** 2025-04-19

**Authors:** Hailu Merga, Teshome Degefa, Zewdie Birhanu, Afework Tadele, Ming-Chieh Lee, Guiyun Yan, Delenasaw Yewhalaw

**Affiliations:** 1 Department of Epidemiology, Institute of Health, Jimma University, Jimma, Ethiopia; 2 School of Medical Laboratory Sciences, Institute of Health, Jimma University, Jimma, Ethiopia; 3 Tropical and Infectious Diseases Research Center, Jimma University, Jimma, Ethiopia; 4 Departement of Health, Behavior, and Society, Faculty of Public Health, Jimma University, Jimma, Ethiopia; 5 Department of Population and Family Health, Faculty of Public Health, Jimma University, Jimma, Ethiopia; 6 Program in Public Health, University of California at Irvine, Irvine, USA

**Keywords:** Urban malaria, Epidemiology, Incidence, Determinants, Scoping review, Sub-Saharan Africa

## Abstract

**Background:**

Malaria control in African cities faces challenges mainly due to unplanned urbanization and the spread of *Anopheles stephensi*. Urbanization is changing malaria dynamics, driven by environmental changes and population growth, with nearly 70% of people projected to live in urban areas by 2050. This scoping review maps the epidemiology of urban malaria in sub-Saharan Africa, identifying research gaps and guiding strategies for control and elimination.

**Methods:**

A structured search across multiple databases was performed using predefined eligibility criteria to select articles. Accordingly, PubMed, Medline EBSCO, Google scholar, Science direct, Cochrane library and grey literature sources were searched for relevant articles. The Joanna Briggs Institute (JBI) guidelines were followed for evidence selection, data extraction, and presentation of findings. Peer-reviewed and gray literature published in English after 2014 that reported on the prevalence, incidence, or risk factors of urban malaria in sub-Saharan Africa was included in the review.

**Results:**

Of the 2459 records identified from various databases, 32 articles were selected for review. A majority of those reviewed studies were community-based studies conducted in urban settings of sub-Saharan African countries. This review found the prevalence of malaria between 0.06% and 58%. This heterogeneity in prevalence is due to differences in diagnostic methods, study design, population characteristics, diagnostic methods, and environmental factors. A majority of those reviewed studies reported the prevalence between 10 and 30% with *Plasmodium falciparum* and *Plasmodium vivax* the dominant species. The review identified key factors associated with urban malaria infection, including socioeconomic status, travel history, prior infection, proximity to water sources, availability of vegetation in the compound, temperature, humidity, livestock ownership, and ITN utilization.

**Conclusion:**

This review found a high prevalence of urban malaria infection in sub-Saharan Africa and there was regional variation. Sociodemographic and socioeconomic status, travel history, ITN utilization, previous history of malaria infection and environmental factors like proximity to water sources, presence of vegetation, temperature, humidity, and livestock ownership were identified as factors associated with urban malaria infection. Hence, there is a need for a comprehensive approach to control urban malaria, including environmental management, improved diagnostics and treatment, socio-economic interventions, and better urban planning.

**Supplementary Information:**

The online version contains supplementary material available at 10.1186/s12936-025-05368-9.

## Background

While malaria is often regarded as a disease primarily affecting the rural poor, it has been a concern in urban areas for centuries. The World Health Organization (WHO) global framework for the response to malaria in urban areas in 2022 indicated that, by 2050, nearly 7 out of 10 people globally will live in cities and other urban settings, majorly expected to occur in Asia and Africa. Although many will benefit from urban living, rapid and unplanned urbanization can create favourable conditions for the spread of infectious diseases, including malaria [1–4].

WHO malaria report 2024 indicated that there were estimated 263 million malaria cases reported globally—an increase of 11 million cases from 2023 report. Sub-Saharan Africa (SSA) bears the highest malaria burden globally and is experiencing significant demographic shifts, with an increasing proportion of its population migrating to urban areas. While urbanization is typically expected to lower malaria transmission, the disease continues to persist in African cities, sometimes at higher rates than in surrounding rural areas [5–7].

The global patterns of disease and mortality, including malaria, will change due to a shift in human population from rural to urban areas [8]. Urbanization has altered health patterns in certain places, making infectious disease risks in urban environments different from those in rural ones [1]. Moreover, unplanned urban expansion, urban agriculture, vegetation, travellers and the spread of mosquito species have a significant impact on the epidemiology of malaria in urban settings [7, 9–12].

Malaria control in urban areas in Africa is also challenged by the recent invasion and expansion of *Anopheles stephensi*, the major vector of urban malaria, as well as urbanization. This vector is well established in Sudan, Djibouti, Somalia, and Ethiopia. Models predicted that the wide spread of the disease’s distribution into cities, where it may cause severe malaria outbreaks among the expanding resident populations [13–16]. In urban areas, *An. stephensi* reproduces in artificial water storage containers and seems to readily adapt to the environment. It also survives in extremely high temperature during the dry season when malaria transmission is low. Moreover, the vector has also exhibited resistance to several classes of insecticide [10, 13–15, 17–19]. If uncontrolled, its spread across the Horn of Africa, combined with rapid and poorly planned urbanization, may increase the risk of urban malaria outbreaks in African cities.

Addressing urban malaria is crucial to meet global health goals like Sustainable Development Goals (SDGs) and WHO’s malaria elimination targets. To prevent the spread of urban malaria, vector control measures, such as insecticide-treated nets (ITNs), indoor residual spraying (IRS), targeted environmental management, and case management and intervention have been used. Depending on the local circumstances, these measures have had differing degrees of success [2].

There is little agreement over what constitutes an urban area or how to define the urbanization process. While size and density are used in certain nations, administrative definitions are used in others. Lack of consensus on the definition of urban has an impact on the reliability and comparability of studies conducted in urban settings. Despite problems with the definition of what constitutes urban, most urban settings in SSA are now viewed as presenting an increased risk of various infectious diseases. Urban malaria refers to the transmission and occurrence of malaria within urban areas and unplanned urbanization is a rapid and haphazard urban expansion characterized by poor housing conditions, inadequate sanitation, and the creation of breeding sites for mosquitoes [1, 8, 20].

Unplanned urbanization increased the number of places where malaria vectors might breed, raising the danger of exposure to mosquito bites and malaria transmission. Urbanization is generally expected to reduce malaria transmission; however, the disease still persists in African cities, in some cases at higher levels than in nearby rural areas [10]. Evidence from sub-Saharan Africa demonstrated that urban transmission differs significantly from district to district within a populous area, and the focused character of transmission necessitates a careful assessment of the risk in different cities [21]. Even though some studies showed that urbanization decreases the morbidity and mortality from malaria [8, 22–24], other recent study showed urbanization as a risk of malaria transmission, especially after the spread of urban malaria vector species [1].

To effectively control malaria, strategies must focus on the specific areas where infections originate. If cases arise from rural regions, interventions should target local vectors, breeding sites, and affected populations. Conversely, if infections occur in urban areas, similar measures must be adapted to the urban context. Consequently, accurate epidemiological data is essential for effective urban malaria control [1].

The study conducted on the impact of urbanization and population density on *Plasmodium falciparum* parasite prevalence rates in Africa demonstrates how urbanization and population density affect the anticipated risk of contracting malaria. This study showed that there is a danger of malaria infection in these heavily populated urban locations throughout Africa [3]. Hence, this scoping review aims to map the prevalence, incidence and risk factors of urban malaria infection in SSA. It aimed to answer the following specific research questions: (1) what is the current prevalence and incidence of urban malaria in SSA? (2) what are the key risk factors associated with urban malaria transmission? (3) how do environmental, socioeconomic, and demographic factors influence urban malaria dynamics? It provides a comprehensive overview of the burden of malaria and identifies gaps in current research and highlights opportunities for targeted interventions. Addressing urban malaria must be addressed in order to meet global health objectives SDGs and the WHO’s malaria elimination targets. This calls for tactics and policy implications that are specific to the particular issues that urban environments present. By addressing the complexity of urban malaria dynamics, this review aims to guide policymakers, researchers, and public health practitioners in designing effective strategies to control and eventually eliminate malaria in rapidly growing urban areas of sub-Saharan Africa.

## Methods

This scoping review was conducted according to the Joanna Briggs Institute (JBI) scoping review guideline and the enhanced framework of the work of Levac, Colquhoun and O’Brien [25]. A scoping review is the most suitable for addressing broad research aims, offering an overview of a field rather than answering specific questions like systematic reviews. It incorporates findings from both qualitative and quantitative studies, regardless of methodology, and maps existing evidence instead of focusing solely on the best available evidence. The Levac and his colleagues and JBI framework suggested five stages that was followed for this review include: identifying the research question, identifying the relevant studies, study selection, charting the data and collating, summarizing and reporting the results [25, 26].

## Inclusion criteria

### Type of participants, concept and context

A comprehensive search strategy was developed in order to identify relevant literature. It was developed based on the ‘PCC’ mnemonic, ‘Population–Concept–Context (PCC)’ framework recommended by the Joanna Briggs Institute for scoping reviews, to construct a clear and meaningful title for the review [26]. This framework was ‘Population (urban residents), Concept (urban malaria) and Context (sub-Saharan Africa)’. The population in this framework was all populations, irrespective of their age and sex, who were living in urban, the concept was urban malaria which included all urban malaria related investigations/studies, irrespective of the type of malaria species, which reported malaria prevalence and risk factors and the context considered was SSA and the countries found in SSA.

## Type of sources

This scoping review considered both observational and analytical studies. It considered also descriptive observational study designs including case series, case reports and descriptive cross-sectional studies. In addition, simple literature review that met the inclusion criteria was considered in this review.

## Information sources and search strategy

The search strategy aimed to locate both published and unpublished studies. A three-step search strategy was utilized in our review. First, an initial search of PubMed, CINAHL (EBSCO), Science direct, Google scholar, and Cochrane library was undertaken to identify articles on the topic. The text words contained in the titles and abstracts of relevant articles, and the index terms used to describe the articles were used to develop a full search strategy (Table 1). The search strategy, including all identified keywords and index terms, was adapted for each included database and/or information source. A search strategy in PubMed data base is attached as supplementary file (S1). The reference lists of all included sources of evidence were screened for additional studies. Only studies published in the English language and those published since 2014 were included. Unpublished articles and gray literature were also searched.

**Table 1:** Search strategy for articles on urban malaria in sub-Saharan Africa

Search strategy item	Search strategy
Data bases	PubMed, Medline EBSCO, Google Scholar, Science direct, Cochrane library
Language filter	English
Time filter	2014–2024
Spatial filter	“Africa south of the Sahara” Or “sub-Saharan Africa” OR"Angola “OR “Benin” OR “Botswana” OR “BURKINA FASO” OR “CABO VERDE” OR “Cameroon” OR “CENTRAL AFRICAN REPUBLIC” OR “Chad” OR “Congo” OR “COTE D’IVOIRE”OR “DEMOCRATIC REPUBLIC OF THE CONGO”OR “Djibouti” OR “EQUATORIAL GUINEA”OR “Eritrea” OR “Eswatini” OR “Ethiopia” OR “Gabon” OR “Gambia” OR “Ghana” OR “Guinea” OR “Kenya” OR “Lesotho” OR “Liberia” OR “Malawi” “Mali” OR “Mauritania” OR “Mozambique” OR “Namibia” OR “Niger” Nigeria"OR “Rwanda” OR “SAO TOME AND PRINCIPE” OR “Senegal” OR “SIERRA LEONE” OR “Somalia” OR ‘SOUTH AFRICA” OR “SOUTH SUDAN” OR “Sudan” OR “Tanzania” OR “Togo” OR “Uganda” OR “Zambia” OR “Zimbabwe”
Keywords	1. “Prevalence” OR “Epidemiology” OR “Cross-Sectional Studies” OR ‘Epidemiology” OR “Incidence” OR “Cohort Studies” 2. “Malaria” OR “malaria, cerebral” OR “Plasmodium falciparum” OR “Plasmodium malariae” OR “Plasmodium ovale” OR “Plasmodium vivax” OR “Acute malaria” 3. “Urban Population” OR “Urbanization” OR “Urban Renewal” OR “Urban Health” OR “town” OR “Cities"
Inclusion criteria	The paper should be: 1. A peer‑reviewed or grey literature 2. A published from 2014 and later 3. Conducted in sub‑Saharan African countries 4. Published in the English language 5. Conducted on urban population or comparison of urban and rural 6. On prevalence or incidence and risk factors/determinants
Exclusion criteria	The paper should meet the following criteria: 1. Focus on rural residents 2. Be conducted in countries outside sub-Saharan Africa 3. Have been published online before the year 2014 4. Include reports, meeting minutes, commentaries, letters to the editor, preprints, systematic review, scoping review, 5. Fall outside the specified variables of interest

## Evidence selection

Following the search, all identified citations were collated and uploaded into the EndNote version 21 reference manager software, and duplications were removed. Then, two independent reviewers screened the title and abstract based on this inclusion criteria. Then, the full texts of the selected citations were included in the reference manager and assessed by independent reviewers. Any disagreements during review were solved through discussion (Table 2).

**Table 2:** Characteristics of all included studies in the scope review of urban malaria in SSA

Author and year of publication	Study design	Sample size	Country	Study area	Key findings	References
Acheampong et al. 2021	Case control study	298	Ghana	Urban and rural	• Urban dwellers exhibited severe forms of malaria compared to rural dwellers with malaria • In both age and gender categories, parasitaemia was significantly higher in urban dwellers than rural dwellers	[27]
Awosolu et al. 2021	Cross- sectional study	300	Nigeria	Urban	• The study showed strong evidence that malaria is still highly prevalent in urban communities • The prevalence of malaria was 55% with mean parasite density of 1814.70 • Age (6–10 years), the presence of streams, living near streams within ≤ 1 km and travel to rural areas were the major risk factors that often increase the odds of malaria infection in this study area	[28]
Ba et al. 2018	Cross- sectional study	4671	Senegal	Urban	• All household members underwent microscopic examination • The prevalence of malaria infection was 0.06% • The prevalence was 0.04% (2/4,671) for *Plasmodium malariae* and 0.02% (1/4,671) for *Plasmodium falciparum*	[29]
Ferrao et al. 2016	Cross- sectional study	490,555	Mozambique	Urban	Weekley maria data from 2006–2014 were collected from Epidemiologic bulletin and found that malaria is high in semi-urban and rural areas than urban	[30]
File and Dinka 2020	Cross- sectional study	2590	Ethiopia	Urban	• The prevalence of malaria during the study period was 3.7% (97/2590) • Adolescents and adults (>/= 15 years of age) were found to be most affected by *Plasmodium vivax* (66%) *and Plasmodium falciparum* (20.5%) • Analysis of the climatological data revealed a rise in environmental temperature and relative humidity during the study that coincides with the increase of malaria cases • History of previous malaria infection was found predictor of malaria infection	[31]
File et al. 2019	Cross- sectional study	6862	Ethiopia	Urban	• A 5 years retrospective data revealed that adolescents and adults (≥ 15 years old) were the most affected by *Plasmodium vivax* 43.5% *and Plasmodium falciparum* 31.7%. It indicated that the burden of *P. vivax* has been increasing over the years compared to falciparum • *Plasmodium vivax* emerged as the dominant species contributing to the malaria burden in the city, showing less seasonal variability	[32]
Georganos et al. 2020	cross-sectional study	–	Uganda and Tanzania	Urban (Kampala and Dar es Salaam)	• This finding indicated, within the urban, that those who were living in informal settlements show higher malaria prevalence compared to those living in planned residential neighborhoods which was • The rapid annual expansion of informal settlements, which often accommodate a significant portion of the urban population, highlights the importance of conducting systematic and consistent malaria surveys in these areas • This study underscores the significance of remote sensing as an epidemiological tool for mapping urban malaria variations across large spatial scales and supporting evidence-based policymaking and control strategies	[33]
Govoetchan et al. 2022	Cross- sectional study	21,008	Benin Republic	Urban and rural	• The prevalence of malaria in urban was 39% and in rural was 36% • Severe cases were more frequent in the urban health facility than in the rural facility • In Urban malaria prevalence was highest among individuals over 15 years old	[34]
Hassen and Dinka 2020	Cross- sectional study	175,423	Ethiopia	Urban	• A retrospective analysis of data from 2012 to 2017 revealed that, the prevalence of malaria was 12.4% of which 49.5% *P. falciparum* and 50.5% *P. vivax*	[35]
Hassen and Dinka 2022	Cross- sectional study	356	Ethiopia	Urban	• Health facility-based study indicated that the prevalence of malaria was 17.13% • Among the malaria-positive patients, 50.8% of them were positive for *Plasmodium vivax*, 45.90% were positive for *Plasmodium falciparum,* and 3.3% had mixed infections • Having insecticide-treated net, houses sprayed with insecticides, and living closer to stagnant water were found to be the factors associated with malaria infection	[36]
Kabaria et al. 2016	Cross- sectional study	–	Tanzania	Urban	• The study showed the existence of malaria risk in urban and which also lacks homogeneity in area • The satellite image also showed heterogeneity in malaria risk within the town which was influenced by varying environmental factors: proximity to dense vegetation, wet/swampy areas and densely built-up areas	[37]
Kazembe and Mathanga 2016	Case control study	767	Malawi	Urban	• The finding showed that visiting rural areas, age of the child, and socio-economic were determinants of urban malaria infection	[38]
Kigozi et al. 2015	Cross- sectional study	1167	Uganda	Urban	• The study found that higher composite urbanicity score in urban was associated with a lower household density of mosquitoes (incidence rate ratio = 0.28) and a lower parasite prevalence (odds ratio, OR = 0.44)	[39]
Kouna et al. 2024	Cross- sectional study	2381	Gabon	Urban, peri-urban and rural	• In urban areas, the overall prevalence of *Plasmodium* spp. infection was 21.6%, the prevalence in semi-rural was 50.6% and the prevalence in rural was 51.2% • Majority were falciparum species • But unequal sample size employed for comparison	[40]
Larson et al. 2021	Cross- sectional study	7564	Malawi	Urban and rural	• The study showed that the prevalence of malaria was 20.6% (slide positive) • The study found that Plasmodium parasitaemia status was strongly associated with distance to lakes, Wealth Index and Use of ITN	[9]
Mathanga et al. 2016	Case control study	473	Malawi	Urban and rural	• Having travelled in the month before testing, electricity in the house, and a higher level of education were associated with malaria infection decreased odds of malaria disease • Travel was the main factor influencing the incidence of malaria illness among urban respondents compared with peri-urban areas	[41]
Merga et al. 2024	Case control study	396	Ethiopia	Urban	• Travel history, presence of eves and holes on the walls, history of malaria diagnosis, owning any livestock, presence of stagnant water, sleeping under bed net the previous night and knowledge on malaria and its prevention were determinants of urban malaria infection	[42]
Mutala et al. 2019	Cross- sectional study	598	Ghana	Urban, peri-urban and rural	• Participants from the rural settlement had the highest malaria prevalence (21.3%) compared to urban (11.8%) and peri-urban areas (13.3%) • However, unequal sample size was taken from each: urban, peri-urban and rural respondents • The peri-urban area had the highest median parasite density • Age was significantly associated with the odds of malaria positivity	[43]
Mwalimu 2019	Cross- sectional study	830	Tanzania	Urban	• Malaria prevalence in the study areas was 4.5% • Low proportion of net ownerships, residing in the households surrounded by mosquito breeding sites and residing in houses with unscreened windows were independently associated with malaria infection	[44]
Ncogo et al. 2015	Cross- sectional study	444	Guinea	Urban	• This study found the high prevalence of malaria infection in rural (58.9%) than urban (33.9%) • But unequal sample size used for this study • Age, having fever in the last 24 months, and ITN utilization were statistically significant variables for the malaria infection in urban	[45]
Ngom and Siegmund 2015	Literature review	–	Cameroon	Urban data	• The findings indicated that the prevalence of urban malaria was strongly influenced by population density and the socio-economic status of the community	[46]
Nyasa et al. 2023	Cross- sectional study	500	Cameroon	Urban and rural	• Malaria prevalence was higher in rural areas 57.6% than urban areas 46.8% • Malaria infection was significantly associated with presence of crops around homes, usage of old LLINs for more than three years and educational status	[47]
Okangba 2019	Literature review	–	sub-Saharan Africa	Urban	• The review showed Lack of education, low income, low wealth, living in poorly constructed houses, and having an occupation in farming may increase risk of Plasmodium infection among people in sub-Saharan Africa	[48]
Paintsil et al. 2024	Cross- sectional study	550	Ghana	Urban	• The finding showed the overall malaria prevalence rate was 7.8% • *Plasmodium falciparum* constituted the majority (97.6%) of the infections, with *Plasmodium ovale* being responsible for only one (2.3%) case • Age and sex were found predictors of malaria infection	[49]
Teka et al. 2023	Cross- sectional study	9,468,970	Ethiopia	Urban	• A retrospective study was done among a total of 9,468,970 malaria cases between 2014 and 2019 • Of these, 1.45 million (15.3%) cases were reported from urban settings • The prevalence of *Plasmodium falciparum was* (67%) *and Plasmodium vivax* (28%) with higher proportion of P. vivax infections in urban areas • The study showed that in 2019, An. stephensi was identified in 17 towns, where over 19,804 malaria cases were reported, with P. falciparum accounting for the majority (56%) of the cases	[50]
Zhou et al. 2016	Cross- sectional study	1434	Ethiopia	Urban	• The prevalence of malaria was 29.8% (with a 1434 suspected malaria cases and 428 confirmed malaria cases) • Among them, 327 (76.4%) cases were *Plasmodium vivax, 97* (22.7%) *were Plasmodium falciparum, and 4 (0.9%) were mixed infection of P. vivax and P. falciparum* • Occupation status, sex, history of malaria illness during the preceding 30 days and history of travel were the determinants of malaria in Jimma town	[51]
Adah et al. 2022	Cross- sectional study	1101	Nigeria	Urban and peri-urban	• The prevalence of malaria was 47% compared to 42% in peri-urban showing that the prevalence was higher in urban than peri-urban	[52]
Arinaitwe et al. 2020	Case control study	567	Uganda	Urban	• Recent overnight, age, staying at relatives’ home, failure to sleep under an ITN during travel and travel to districts that had not received IRS were determinants of malaria infection	[53]
Savi et al. 2022	–	–	Ghana	Urban	• The study found that the prevalence of malaria was impacted by seasonality, but the trend of the seasonal signature is not noticeable in urban and peri-urban areas • While urban districts have a slightly lower prevalence, there are still pockets with higher rates within these regions • The areas of high prevalence of malaria in urban were linked to proximity to water bodies and waterways	[54]
García et al. 2023	Cross sectional	25,920	Bioko Island	Urban and peri-urban	• This survey compared the malaria prevalence between 2015 and 2018 by comparing urban and rural population included in the survey • The prevalence of urban malaria was 12% in 2015 and 10.9% in 2018. The prevalence reported from urban in 2018 was higher than the prevalence reported from rural (10.1%) in the same year • Age, sex, travel history and ITN utilization were determinants of urban malaria	[55]
Molla et al. 2024	Cross sectional	504	Ethiopia	Urban and rural	• The prevalence of urban malaria was 29.6% • For all seasons, malaria infection was significantly higher in the urban setting • Residence, anemia status, mosquito net utilization and distance from mosquito breeding places were determinants of urban malaria	[56]
Chiziba et al. 2024	Cross-sectional study	14,139	Nigeria	Urban	• The study reported malaria positivity rate by clusters (988 clusters) from 2010 to 2021 • Vegetation index, population density, age, wealth index, educational level	[57]

## Data extraction and analysis

Three independent reviewers extracted the data included in the scoping review using the tool developed by the reviewers. The data extracted includes specific details about the participants, concept, context, study methods and key findings relevant to the review question/s. Finally, data were extracted on structured data extraction form and presented using tables. JBI Template study details, characteristics and results extraction instrument was used.

## Results

**Fig. 1:**
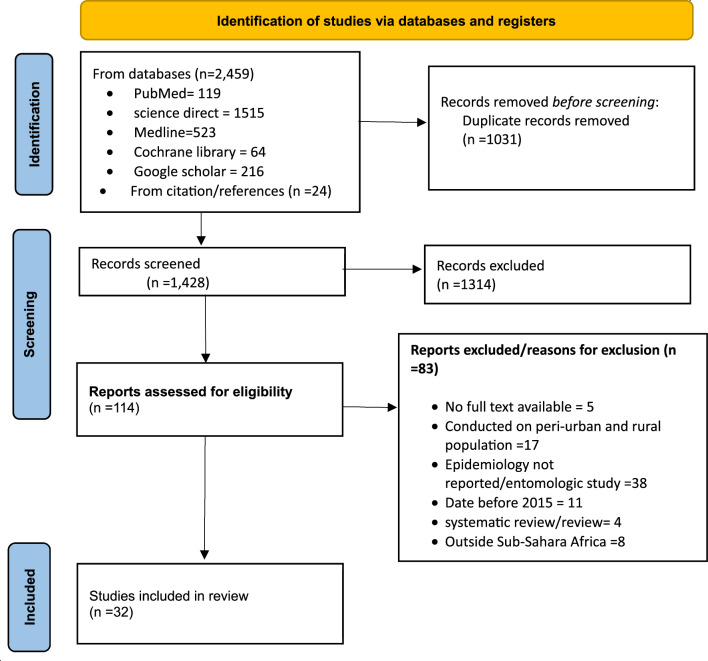
PRISMA flow chart diagram for scoping review on Urban malaria Epidemiology in Sub-Saharan Africa (SSA)

The initial literature search resulted in a total of 2437 from different databases as shown on PRISMA below. In addition, 24 papers were identified through citation and reference checking. After removing 1031 duplications, 1428 were screened for title and abstract. Furthermore, 1314 records were excluded for not meeting the inclusion criteria. Overall, 114 full-text articles were deemed eligible for further screening. Ultimately, 32 full-text articles were included in the thematic analysis and synthesis (Fig. 1).

## Characteristics of reviewed studies

**Fig. 2:**
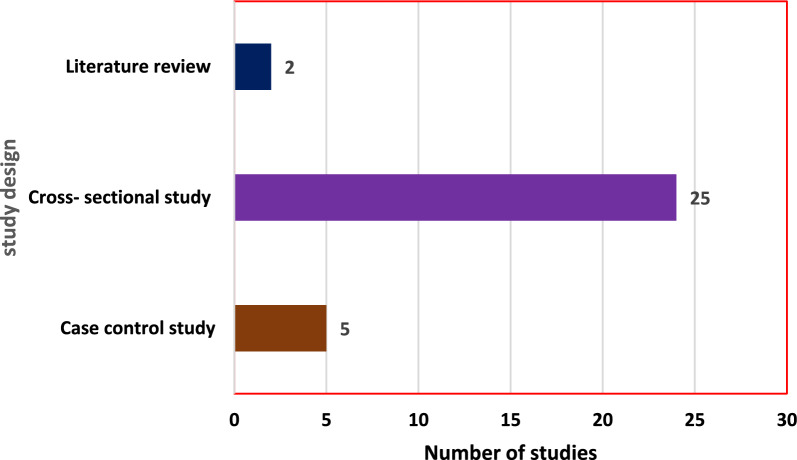
Study design of reviewed studies in sub-Saharan Africa

**Fig. 3:**
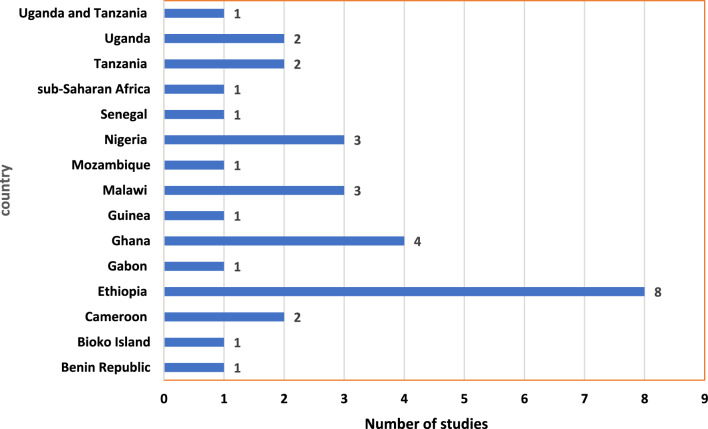
Countries where reviewed studies were conducted in sub-Saharan Africa

**Fig. 4:**
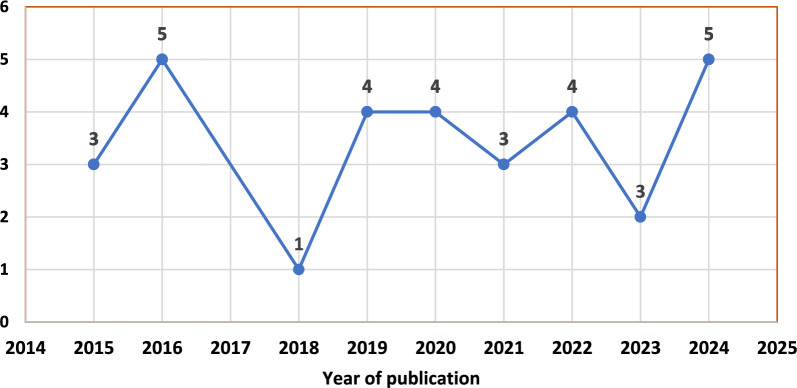
The year reviewed studies were published online

A majority of those reviewed studies were community-based studies conducted in urban settings of SSA countries. This review indicated that majority of the study design of the reviewed studies were cross sectional studies (24) followed by case control studies (5) (Fig. 2). Among studies included in the review eight were from Ethiopia and four were from Ghana (Fig. 3). Among those who reported species, majority of them reported *P. falciparum* as the dominant species. Most of those studies were published online after 2018 (Fig. 4).

## Prevalence of urban malaria in sub-Saharan Africa

This study reviewed the prevalence of urban malaria in sub-Saharan Africa. Some of these studies were conducted in urban areas only, and some of them were comparing the prevalence or incidence of malaria in urban and rural areas. The findings of included studies in the review indicated that sociodemographic and environmental variability significantly influenced how urban malaria spreads. The review indicated that the study encompassed a wide range of population characteristics and methodologies. For instance, some studies focused on children, while others included all patients visiting health facilities regardless of age. Additionally, certain studies focused on febrile patients attending health facilities; some studies diagnosed all household members, others utilized retrospective data from health facilities, and a few employed satellite imagery and associated data. To report prevalence, the majority of studies used microscopy for laboratory diagnosis, while a few relied solely on RDT, others combined RDT, microscopy, and PCR, and some used microscopy and PCR. Accordingly, this review found the prevalence of malaria between 0.06% in Senegal [29] and 55% in Nigeria [28]. Majority of the studies reported the prevalence between 10 and 30% [9, 35, 36, 40, 43, 50, 51, 55, 56]. Some studies reported high prevalence of malaria infection 55%, 39%, 33.9%, 46.8% and 47% [28, 34, 45, 47, 52] while few studies reported less than 5% [29, 31, 44]. On the other hands, though unequal sample size was employed, some of the studies that were conducted among urban and rural residents found that the prevalence of malaria was higher in urban compared rural residents [34, 52, 55, 56]. However, some studies also found the higher prevalence in rural than urban [30, 40, 43]. With regards to the malaria species, even though some of the studies didn`t report the species, some of those reviewed studies showed that *P. falciparum* is the dominant species in urban settings [40, 49, 50], while other studies also reported *Plasmodium vivax* [32, 35, 36, 51] as a dominant species. Among those reviewed studies, *Plasmodium malariae* was also reported from Senegal [29].

## Risk factors of urban malaria in sub-Saharan Africa

### Socio- sociodemographic economic and socio-economic factors

There are many studies that assess the determinants of urban malaria majorly using cross sectional study and case control study designs. The finding from Ghana showed that urban dwellers, in both age and sex categories, exhibited more severe forms of malaria compared to rural dwellers. The finding revealed that urban dwellers were more prone to severe malaria while rural dwellers tended to have more measured immune responses against malaria infection [27]. In addition, most studies found age as the determinant of malaria infection [28, 31, 34, 38, 43, 45, 49, 53, 55, 57]. Similarly, in some of the reviewed studies, sex had a statistically significant association with urban malaria infection [27, 49, 52, 55]On the other hand, among those reviewed studies, a simple literature review from sub-Saharan Africa [48], finding from Malawi and Nigeria reported wealth index or income had an influence on malaria infection in urban settings [38, 57]. Similarly, a simple literature review from Cameroon indicated that the prevalence of urban malaria is strongly influenced by population density and the socio-economic status of the community [46, 57]. Another study from this country also reported educational status as the determinant of malaria infection [47]. On the other hand, a study from Malawi indicated that having electricity in the house and a higher level of educational status decreased odds of malaria disease [41]. A finding from southern Ethiopia also revealed occupation status as the determinants of malaria infection [51].

## Travel history

Some articles included in the review reported population mobility or travel history as the main determinants of urban malaria infection. The community-based cross-sectional study from Nigeria and Bioko Island showed having history of travel increased the odds of malaria infection [28, 55]. Similarly, among studies included in this review, a study from Malawi, Uganda and two studies from Ethiopia reported travel history as the main factor influencing the incidence of malaria illness among urban respondents [41, 42, 51, 53].

## History of malaria infection, ITN utilization and environmental factors

Some of the reviewed studies showed history of previous malaria infection as the predictor of urban malaria infection [31, 42, 51]. Among environmental factors, the study from Nigeria and Ethiopia reported that the presence of streams and living near streams increase the odds of malaria infection [28, 56]. Similarly, an article included in the review from Ethiopia showed the rise in environmental temperature and relative humidity during the study that coincides with the increase of malaria cases, since it creates favourable mosquito breeding for malaria transmission in the city [31]. The finding from Uganda and Tanzania reported that, within the urban area, those who were living in informal settlements showed higher malaria prevalence compared to those living in planned residential neighbourhoods. Similarly, variation in malaria risk within the town was shown to be influenced by varying environmental factors like proximity to dense vegetation, wet/swampy areas and densely built-up areas [33, 37]. Similarly, studies included in the review from Ethiopia reported that houses sprayed with insecticides, living closer to stagnant water, presence of eves and holes on the walls, owning any livestock and sleeping under bed net the previous night were the determinants of urban malaria infection [36, 42]. Use of ITN was found to be predictor of urban malaria in Malawi, Bioko Island and Ethiopia [9, 55, 56]. In Uganda, low proportion of net ownership, residing in the households surrounded by mosquito breeding sites and residing in houses with unscreened windows were independently associated with malaria infection in urban setting [44]. The studies included in this review from Cameroon showed that malaria infection was significantly associated with the presence of crops around homes and usage of old long-lasting insecticidal nets (LLINs) for more than three years [47].

## Discussion

This review highlights the high burden of urban malaria infections in sub-Saharan African countries, examining its prevalence and incidence. The included studies varied in scope, with some exclusively focused on urban settings and others comparing urban and rural areas, encompassing diverse population groups and methodologies. The prevalence of urban malaria varied widely, ranging from 0.06% to 55%, may be due to differences in study settings, population characteristics, diagnostic methods, and environmental factors influencing malaria transmission. Moreover, the review also identified multiple risk factors associated with urban malaria infection, including sociodemographic and socioeconomic factors, travel history, ITN utilization, prior history of malaria infection, and environmental factors.

The high prevalence of urban malaria in sub-Saharan Africa may be attributed to a mix of environmental, demographic, and socio-economic factors. Rapid urbanization has led to poor infrastructure, such as inadequate drainage systems, which create breeding grounds for mosquitoes. High population density increases human-vector interactions, while migration from rural to urban areas introduces malaria cases. Challenges in implementing preventive measures, such as inconsistent ITN usage, and climatic factors like urban heat islands further exacerbate the problem [1, 58]. For instance, studies that were facility-based reported high prevalence of malaria [28, 34]. This might be due to the likelihood of detecting malaria parasites maybe higher in health facility samples, as patients typically present with signs and symptoms, potentially leading to an overestimation of malaria prevalence in such settings. Besides, microscopy may produce a high number of false negatives despite its gold standard because of the lack of experienced microscopists and the effect of self-medication which is common in SSA [59, 60]. On the other hand, some studies used unequal sample sizes, particularly in comparisons of malaria prevalence between urban and rural areas [34, 52]. This disparity can affect the accuracy of prevalence estimates due to the imbalance in the sample denominators. However, few studies also reported the higher prevalence in rural than urban area [30, 40, 43]. However, none of these studies used equal sample sizes, which can influence the magnitude of prevalence estimates.

In this review, many studies have assessed the determinants of urban malaria infection, identifying various factors that may vary based on the study design and the population included. These differences in findings could be attributed to the methodologies used and the specific characteristics of the study population. In most of the reviewed studies, sociodemographic variables like age and sex were commonly found as determinants of malaria because they directly influence susceptibility. In some of the studies, the respondents were children and pregnant mothers. Additionally, sex differences may relate to varying exposure levels, cultural practices, and access to healthcare. These factors are often included in studies to understand how demographic characteristics impact malaria transmission and prevalence [40, 61, 62].

In this review, travel history was found to be a key determinant of urban malaria infection in many studies because individuals who travel to or from malaria-endemic areas can introduce the parasite into urban environments. This is particularly important in cities where the local vector may not traditionally be present, or where the population has less immunity to malaria. Migrants or travellers can significantly contribute to the spread of malaria by bringing in new cases, which can then amplify urban transmission [6, 21, 63].

A history of previous malaria infection was found a predictor of urban malaria in some studies included in this review [31, 42] This might be due to the relapse nature of *P. vivax* and recrudescence nature of *P. falciparum*, as both these species were reported in this review, although *P. falciparum* was the dominant species. *Plasmodium vivax* is well known for relapse due to the survival of latent liver forms known as hypnozoites which may remain in the liver from a few months to years following the initial infection, and if conditions are favourable, they will become active and induce relapse of malaria despite the apparent recovery of the individual [64–66]. On the other hand, *P. falciparum* can cause recrudescence, where parasitaemia reapers due to incomplete treatment or partial immune response. This means that if a person with P. falciparum malaria does not fully clear the infection, the parasites can reemerge, leading to the disease returning [67–69].

Among environmental factors, many studies included in the review reported that the presence of streams and living near streams or swampy areas, rise in environmental temperature and relative humidity, dense vegetation, and densely built-up areas, living closer to stagnant water, presence of eaves and holes on the walls, and owning any livestock. These factors create favourable environments for *Anopheles* mosquitoes, promoting their survival and facilitating the transmission of the *Plasmodium* parasite. Stagnant water near homes also provides ideal breeding sites for mosquito larvae, increasing their population and the risk of malaria transmission. Similarly, higher temperatures accelerate the development of the malaria parasite inside mosquitoes, enabling faster transmission while also increasing mosquito activity and reproduction. However, extreme heat may negatively impact mosquito survival, potentially limiting transmission in very hot climates. Poor urban planning often creates stagnant water sites, such as open drains or construction areas, which serve as mosquito breeding grounds and increase transmission risk in densely populated areas. Additionally, open eaves and cracks in walls provide easy entry points for mosquitoes, exposing residents to more bites indoors. On the other hand, livestock attract mosquitoes by emitting carbon dioxide and odors, drawing them closer to human dwellings. This proximity increases the chances of mosquitoes biting both humans and animals, facilitating malaria transmission. In this review, low proportion of net ownerships and utilization were independently associated with malaria infection in urban setting. This is due to ITN ownership and utilization are being crucial in reducing malaria transmission [1, 2, 36, 37, 44, 58, 70–72].

## Limitations

The limitations of this scoping review include the use of different laboratory diagnostic methods, which may introduce inconsistencies in the data and affect the comparability of prevalence and risk factors for malaria infection. To address this, future reviews could standardize diagnostic criteria or subgroup analysis by method. The review also included studies with different designs and populations, which may complicate data synthesis. Grouping studies by design type or population could improve clarity. Furthermore, limiting the review to English-language studies may restrict its comprehensiveness, and expanding it to include non-English studies could enrich the findings.

## Conclusion

This review found that there is a high prevalence of urban malaria infections in sub-Saharan Africa, with significant variability observed across studies. Differences in study settings, population characteristics, diagnostic methods, and environmental factors likely contribute to this variability. Additionally, the review identified several risk factors for urban malaria, including sociodemographic and socioeconomic factors, travel history, ITN ownership and utilization, history of malaria infections, and environmental factors. This review underscores the complexity of controlling urban malaria, which requires a comprehensive approach that includes environmental management, improved diagnostics and treatment, socio-economic interventions, and better urban planning to reduce mosquito breeding sites and human exposure.

## Supplementary Information

Supplementary Material 1. Search strategy in PubMed data base.

## Data Availability

Data is provided within the manuscript or supplementary information files.

## Abbreviations

ICMER: International center of excellence for malaria research
ITN: Insecticide-treated net
JBI: Joanna briggs institute
LLIN: Long-lasting insecticidal nets
NIH: National institute of health
SSA: Sub-Saharan Africa
WHO: World Health Organization
