# Supplementary material for: Urban malaria in sub-Saharan Africa: a scoping review of epidemiologic studies
Source: Malar J. 2025 Apr 19;24:131. doi: 10.1186/s12936-025-05368-9 (PMC12009534; doi:10.1186/s12936-025-05368-9)
Supplement: Supplementary file 1 — Supplementary Material 1. Search strategy in PubMed data base. [file 12936_2025_5368_MOESM1_ESM.docx]

**Table 2: Search strategy in PubMed data base**

| Search number | Query | Search Details | Results |
| --- | --- | --- | --- |
| 1 | ("Prevalence"[Mesh] OR "Epidemiology"[Mesh] OR "Cross-Sectional Studies"[Mesh] OR "epidemiology" [Subheading]) OR ( "Incidence"[Mesh] OR "Cohort Studies"[Mesh] ) | "Prevalence"[MeSH Terms] OR "Epidemiology"[MeSH Terms] OR "Cross-Sectional Studies"[MeSH Terms] OR "Epidemiology"[MeSH Subheading] OR "Incidence"[MeSH Terms] OR "Cohort Studies"[MeSH Terms] | 4,972,555 |
| 2 | "Malaria"[Mesh] OR "Malaria, Cerebral"[Mesh] OR "Malaria, Vivax"[Mesh] OR "Malaria, Falciparum"[Mesh] OR "Acute malaria" [Supplementary Concept] | "Malaria"[MeSH Terms] OR "malaria, cerebral"[MeSH Terms] OR "malaria, vivax"[MeSH Terms] OR "malaria, falciparum"[MeSH Terms] OR "Acute malaria"[Supplementary Concept] | 77,176 |
| 3 | "Urban Population"[Mesh] OR "Urbanization"[Mesh] OR "Urban Renewal"[Mesh] OR "Urban Health"[Mesh] OR "Cities"[Mesh] | "Urban Population"[MeSH Terms] OR "Urbanization"[MeSH Terms] OR "Urban Renewal"[MeSH Terms] OR "Urban Health"[MeSH Terms] OR "Cities"[MeSH Terms] | 229,897 |
| 4 | ANGOLA OR BENIN OR BOTSWANA OR "BURKINA FASO" OR "CABO VERDE" OR CAMEROON OR "CENTRAL AFRICAN REPUBLIC"OR CHAD OR CONGO OR "COTE D'IVOIRE" OR "DEMOCRATIC REPUBLIC OF THE CONGO" OR DJIBOUTI OR "EQUATORIAL GUINEA" OR ERITREA OR ESWATINI OR ETHIOPIA OR GABON OR GAMBIA OR GHANA OR GUINEA OR GUINEA-BISSAU OR KENYA OR LESOTHO OR LIBERIA OR MALAWI OR MALI OR MAURITANIA OR MOZAMBIQUE OR NAMIBIA OR NIGER OR NIGERIA OR RWANDA OR "SAO TOME AND PRINCIPE"OR SENEGAL OR "SIERRA LEONE" OR SOMALIA OR "SOUTH AFRICA" OR "SOUTH SUDAN" OR SUDAN OR TANZANIA OR TOGO OR UGANDA OR ZAMBIA OR ZIMBABWE OR "africa south of the sahara"[MeSH Terms] OR sub saharan africa[Text Word] | "angola"[MeSH Terms] OR "angola"[All Fields] OR "angola s"[All Fields] OR ("benin"[MeSH Terms] OR "benin"[All Fields] OR "benin s"[All Fields]) OR ("botswana"[MeSH Terms] OR "botswana"[All Fields] OR "botswana s"[All Fields]) OR "BURKINA FASO"[All Fields] OR "CABO VERDE"[All Fields] OR ("cameroon"[MeSH Terms] OR "cameroon"[All Fields] OR "cameroons"[All Fields] OR "cameroon s"[All Fields]) OR "CENTRAL AFRICAN REPUBLIC"[All Fields] OR ("chad"[MeSH Terms] OR "chad"[All Fields]) OR ("congo"[MeSH Terms] OR "congo"[All Fields]) OR "COTE D'IVOIRE"[All Fields] OR "DEMOCRATIC REPUBLIC OF THE CONGO"[All Fields] OR ("djibouti"[MeSH Terms] OR "djibouti"[All Fields]) OR "EQUATORIAL GUINEA"[All Fields] OR ("eritrea"[MeSH Terms] OR "eritrea"[All Fields]) OR ("eswatini"[MeSH Terms] OR "eswatini"[All Fields]) OR ("ethiopia"[MeSH Terms] OR "ethiopia"[All Fields] OR "ethiopia s"[All Fields]) OR ("gabon"[MeSH Terms] OR "gabon"[All Fields]) OR ("gambia"[MeSH Terms] OR "gambia"[All Fields] OR "gambia s"[All Fields]) OR ("ghana"[MeSH Terms] OR "ghana"[All Fields] OR "ghana s"[All Fields]) OR ("guinea"[MeSH Terms] OR "guinea"[All Fields] OR "guinea s"[All Fields] OR "guineas"[All Fields]) OR ("guinea bissau"[MeSH Terms] OR "guinea bissau"[All Fields] OR ("guinea"[All Fields] AND "bissau"[All Fields]) OR "guinea bissau"[All Fields]) OR ("kenya"[MeSH Terms] OR "kenya"[All Fields] OR "kenya s"[All Fields]) OR ("lesotho"[MeSH Terms] OR "lesotho"[All Fields] OR "lesotho s"[All Fields]) OR ("liberia"[MeSH Terms] OR "liberia"[All Fields] OR "liberia s"[All Fields]) OR ("malawi"[MeSH Terms] OR "malawi"[All Fields] OR "malawi s"[All Fields]) OR ("mali"[MeSH Terms] OR "mali"[All Fields]) OR ("mauritania"[MeSH Terms] OR "mauritania"[All Fields]) OR ("mozambique"[MeSH Terms] OR "mozambique"[All Fields] OR "mozambique s"[All Fields]) OR ("namibia"[MeSH Terms] OR "namibia"[All Fields] OR "namibia s"[All Fields]) OR ("niger"[MeSH Terms] OR "niger"[All Fields]) OR ("nigeria"[MeSH Terms] OR "nigeria"[All Fields] OR "nigeria s"[All Fields]) OR ("rwanda"[MeSH Terms] OR "rwanda"[All Fields] OR "rwanda s"[All Fields]) OR "SAO TOME AND PRINCIPE"[All Fields] OR ("senegal"[MeSH Terms] OR "senegal"[All Fields] OR "senegal s"[All Fields]) OR "SIERRA LEONE"[All Fields] OR ("somalia"[MeSH Terms] OR "somalia"[All Fields] OR "somalia s"[All Fields]) OR "SOUTH AFRICA"[All Fields] OR "SOUTH SUDAN"[All Fields] OR ("sudan"[MeSH Terms] OR "sudan"[All Fields] OR "sudans"[All Fields] OR "sudan s"[All Fields]) OR ("tanzania"[MeSH Terms] OR "tanzania"[All Fields] OR "tanzania s"[All Fields]) OR ("togo"[MeSH Terms] OR "togo"[All Fields]) OR ("uganda"[MeSH Terms] OR "uganda"[All Fields] OR "uganda s"[All Fields]) OR ("zambia"[MeSH Terms] OR "zambia"[All Fields] OR "zambia s"[All Fields]) OR ("zimbabwe"[MeSH Terms] OR "zimbabwe"[All Fields] OR "zimbabwe s"[All Fields]) OR "africa south of the sahara"[MeSH Terms] OR "sub saharan africa"[Text Word] | 734,435 |
| 5 | #1 AND #2 AND #3 AND #4 | (("Prevalence"[MeSH Terms] OR "Epidemiology"[MeSH Terms] OR "Cross-Sectional Studies"[MeSH Terms] OR "Epidemiology"[MeSH Subheading] OR ("Incidence"[MeSH Terms] OR "Cohort Studies"[MeSH Terms])) AND ("Malaria"[MeSH Terms] OR "malaria, cerebral"[MeSH Terms] OR "malaria, vivax"[MeSH Terms] OR "malaria, falciparum"[MeSH Terms] OR "Acute malaria"[Supplementary Concept]) AND ("Urban Population"[MeSH Terms] OR "Urbanization"[MeSH Terms] OR "Urban Renewal"[MeSH Terms] OR "Urban Health"[MeSH Terms] OR "Cities"[MeSH Terms]) AND ("angola"[MeSH Terms] OR "angola"[All Fields] OR "angola s"[All Fields] OR ("benin"[MeSH Terms] OR "benin"[All Fields] OR "benin s"[All Fields]) OR ("botswana"[MeSH Terms] OR "botswana"[All Fields] OR "botswana s"[All Fields]) OR "BURKINA FASO"[All Fields] OR "CABO VERDE"[All Fields] OR ("cameroon"[MeSH Terms] OR "cameroon"[All Fields] OR "cameroons"[All Fields] OR "cameroon s"[All Fields]) OR "CENTRAL AFRICAN REPUBLIC"[All Fields] OR ("chad"[MeSH Terms] OR "chad"[All Fields]) OR ("congo"[MeSH Terms] OR "congo"[All Fields]) OR "COTE D'IVOIRE"[All Fields] OR "DEMOCRATIC REPUBLIC OF THE CONGO"[All Fields] OR ("djibouti"[MeSH Terms] OR "djibouti"[All Fields]) OR "EQUATORIAL GUINEA"[All Fields] OR ("eritrea"[MeSH Terms] OR "eritrea"[All Fields]) OR ("eswatini"[MeSH Terms] OR "eswatini"[All Fields]) OR ("ethiopia"[MeSH Terms] OR "ethiopia"[All Fields] OR "ethiopia s"[All Fields]) OR ("gabon"[MeSH Terms] OR "gabon"[All Fields]) OR ("gambia"[MeSH Terms] OR "gambia"[All Fields] OR "gambia s"[All Fields]) OR ("ghana"[MeSH Terms] OR "ghana"[All Fields] OR "ghana s"[All Fields]) OR ("guinea"[MeSH Terms] OR "guinea"[All Fields] OR "guinea s"[All Fields] OR "guineas"[All Fields]) OR ("guinea bissau"[MeSH Terms] OR "guinea bissau"[All Fields] OR ("guinea"[All Fields] AND "bissau"[All Fields]) OR "guinea bissau"[All Fields]) OR ("kenya"[MeSH Terms] OR "kenya"[All Fields] OR "kenya s"[All Fields]) OR ("lesotho"[MeSH Terms] OR "lesotho"[All Fields] OR "lesotho s"[All Fields]) OR ("liberia"[MeSH Terms] OR "liberia"[All Fields] OR "liberia s"[All Fields]) OR ("malawi"[MeSH Terms] OR "malawi"[All Fields] OR "malawi s"[All Fields]) OR ("mali"[MeSH Terms] OR "mali"[All Fields]) OR ("mauritania"[MeSH Terms] OR "mauritania"[All Fields]) OR ("mozambique"[MeSH Terms] OR "mozambique"[All Fields] OR "mozambique s"[All Fields]) OR ("namibia"[MeSH Terms] OR "namibia"[All Fields] OR "namibia s"[All Fields]) OR ("niger"[MeSH Terms] OR "niger"[All Fields]) OR ("nigeria"[MeSH Terms] OR "nigeria"[All Fields] OR "nigeria s"[All Fields]) OR ("rwanda"[MeSH Terms] OR "rwanda"[All Fields] OR "rwanda s"[All Fields]) OR "SAO TOME AND PRINCIPE"[All Fields] OR ("senegal"[MeSH Terms] OR "senegal"[All Fields] OR "senegal s"[All Fields]) OR "SIERRA LEONE"[All Fields] OR ("somalia"[MeSH Terms] OR "somalia"[All Fields] OR "somalia s"[All Fields]) OR "SOUTH AFRICA"[All Fields] OR "SOUTH SUDAN"[All Fields] OR ("sudan"[MeSH Terms] OR "sudan"[All Fields] OR "sudans"[All Fields] OR "sudan s"[All Fields]) OR ("tanzania"[MeSH Terms] OR "tanzania"[All Fields] OR "tanzania s"[All Fields]) OR ("togo"[MeSH Terms] OR "togo"[All Fields]) OR ("uganda"[MeSH Terms] OR "uganda"[All Fields] OR "uganda s"[All Fields]) OR ("zambia"[MeSH Terms] OR "zambia"[All Fields] OR "zambia s"[All Fields]) OR ("zimbabwe"[MeSH Terms] OR "zimbabwe"[All Fields] OR "zimbabwe s"[All Fields]) OR "africa south of the sahara"[MeSH Terms] OR "sub saharan africa"[Text Word])) AND (y_10[Filter]) | 119 |
